# Primary presacral neuroendocrine tumor associated with imperforate anus

**DOI:** 10.1186/1477-7819-5-115

**Published:** 2007-10-11

**Authors:** Tad Kim, Stephen R Grobmyer, Chen Liu, Steven N Hochwald

**Affiliations:** 1 Division of Surgical Oncology, University of Florida, College of Medicine, Gainesville, FL 32610, USA; 2 Departments of Pathology, Immunology and Laboratory Medicine, University of Florida, College of Medicine, Gainesville, FL 32610, USA

## Abstract

**Background:**

Presacral masses are unusual growths that have a limited differential diagnosis, typically not including neuroendocrine tumors (NETs). Classically, NETs are well-differentiated gastroenteropancreatic tumors of probable benign behavior. These tumors are associated with a typical morphologic pattern and involve the distal colon, rectum, and genitourinary tract; they are considered less aggressive, frequently asymptomatic, and rarely cause carcinoid syndrome, even when metastatic. Neuroendocrine tumors of the presacral region are extremely rare and few have been described in the literature. They have not been previously reported as being associated with imperforate anus.

**Case presentation:**

We present an interesting case of a woman with a history of imperforate anus that was found to have a primary neuroendocrine tumor of the presacral region with no rectal wall involvement.

**Conclusion:**

We argue that this is a primary gastroenteropancreatic neuroendocrine tumor which likely originated from cells of hindgut origin that underwent an abnormal migration during embryonic development.

## Background

Neuroendocrine tumors are rare with an incidence rate of two to five per 100,000, with a higher incidence rate for African-American men and women. The age distribution ranges from the second to the ninth decade, with peak incidence between the ages of 50 and 70. They are the most common neuroendocrine tumor of the gastrointestinal tract. Around 55% are located in the gastrointestinal tract and 30% in the bronchopulmonary system. In the gastrointestinal tract, they tend to arise most commonly in the small intestine (45%), followed by rectum (20%), appendix (16%), colon (11%), and stomach (7%) [1].

Neuroendocrine tumors of the presacral region are rare and usually represent direct extension or metastasis from primary rectal tumors. Only a handful of primary presacral NETs have been reported in the literature and are felt presumably to derive from hindgut rests, especially given their histopathologic similarity with primary rectal NETs [2]. We report a rare case of a primary presacral NET in a patient with a history of imperforate anus, which is associated with abnormalities in hindgut cell migration and explains the predisposition to develop a classic hindgut-like gastroenteropancreatic NET in this region.

## Case presentation

**Figure 1:**
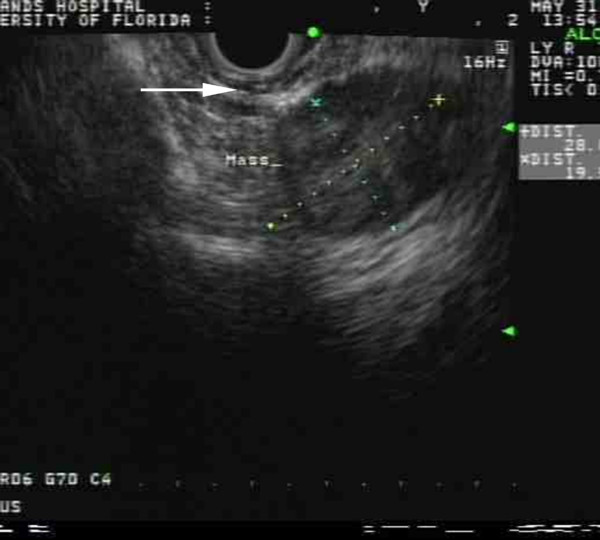
**EUS image of rectal wall and presacral space**. Arrow indicates muscularis propria of the rectum. Tumor is outside this layer of the bowel wall.

A 58 year old African-American female reported symptoms of abdominal pain and diarrhea. Physical examination including abdominal, vaginal, cervical and rectal exam were not contributory. She was found on computerized tomography scan of the abdomen/pelvis to have a 3.2 cm heterogeneous mass anterior to the coccyx, with central hypodensity and peripheral rim enhancement; the mass appeared separate from the posterior rectal wall. The pelvic organs were unremarkable, as no other abnormalities were identified on CT scan. Subsequent CT-guided biopsy and pathology was consistent with a neuroendocrine tumor. Colonoscopy was negative for primary tumor of the rectum. Transanal endoscopic ultrasound confirmed a heterogeneous and hypoechoic mass at 13 cm from the anal verge, clearly separate from the posterior rectal wall, with no discernible lymphadenopathy (Figure 1). The posterior vaginal wall was visualized and unremarkable. Somatostatin receptor scintigraphy scan (SSRS) revealed uptake in the presacral mass compatible with neuroendocrine tumor, but no other sites of disease.

**Figure 2:**
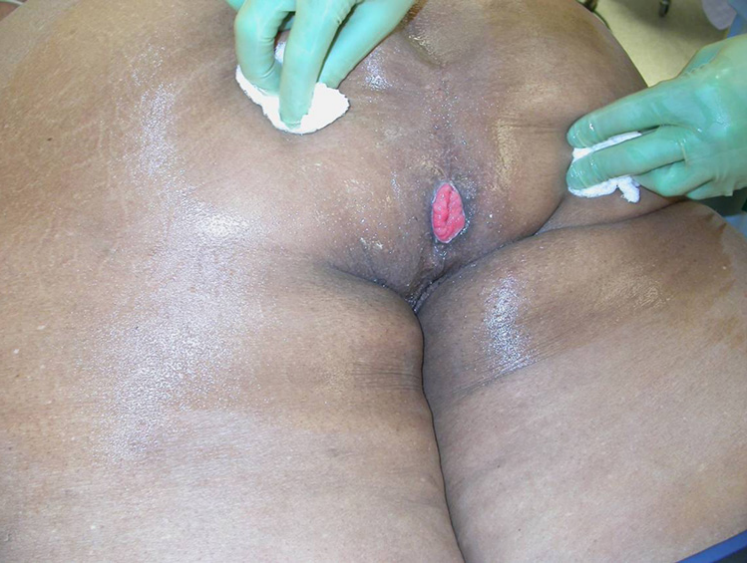
Imperforate anus with neoanus.

The patient had a history of imperforate anus, having lived with a colostomy up until ten years of age. At that time she underwent two coloanal pull through procedures through both an abdominal and transsacral approach with creation of a neoanus (Figure 2). Prior to our evaluation, her bowel function was described as having fecal continence with occasional loose stools and irregular bowel movements. Of note, she did not have any wheezing, flushing, pellagra, or other symptomatology to meet a constellation typical of the "carcinoid syndrome". Her abdominal pain resolved prior to any treatment for her presacral mass and was felt not related to her NET. Therefore, her NET was considered an incidental finding.

**Figure 3:**
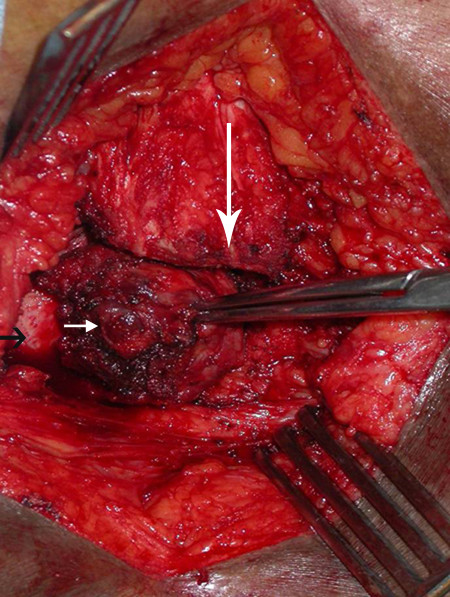
**Transsacral approach to presacral mass**. Intraoperative picture showing divided sacral bone (long white arrow), tumor mass (short white arrow) and underlying rectal wall (black arrow).

**Figure 4:**
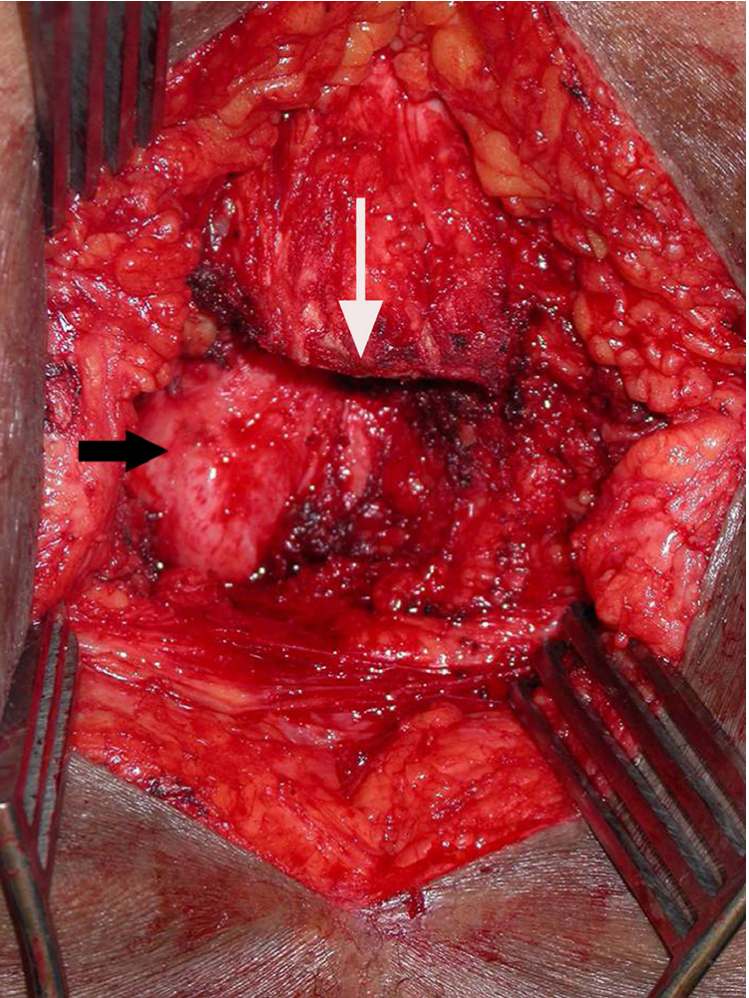
**Operative field following resection**. Operative field following resection of the tumor, showing divided sacral bone (white arrow) and underlying rectal wall (black arrow).

**Figure 5:**
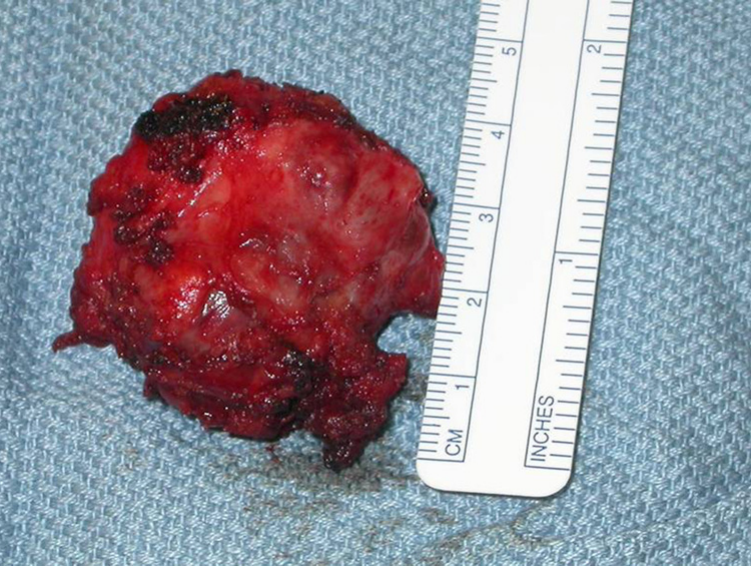
**Tumor mass**. Resected tumor mass.

At operation, she underwent transsacral resection of the NET with partial removal of the sacrum and removal of the entire coccyx and mass (Figures 3, 4 and 5). The surgery was complicated by the presence of scar tissue from previous coloanal pull through procedures. A vertical incision over the sacrum and coccyx was performed. The subcutaneous tissue was divided down to the sacral bone. The tumor appeared to extend in the presacral plane up to about the S4-5 level. Thus, the sacral bone was divided at the S4 level and then the presacral area was entered and dissection continued in this plane. Notably, the tumor was slightly adherent to perirectal fat, but not invading or emanating from the rectal wall. The tumor was dissected away without difficulty from the posterior rectal wall and neoanus.

Postoperatively, the patient did well and was discharged without complications. She had no new symptoms of incontinence, rectal, or back pain. Her bowel habits and loose stools remained at her pre-operative baseline. There was no evidence of recurrence of disease after 10 months of follow up.

### Pathology

**Figure 6:**
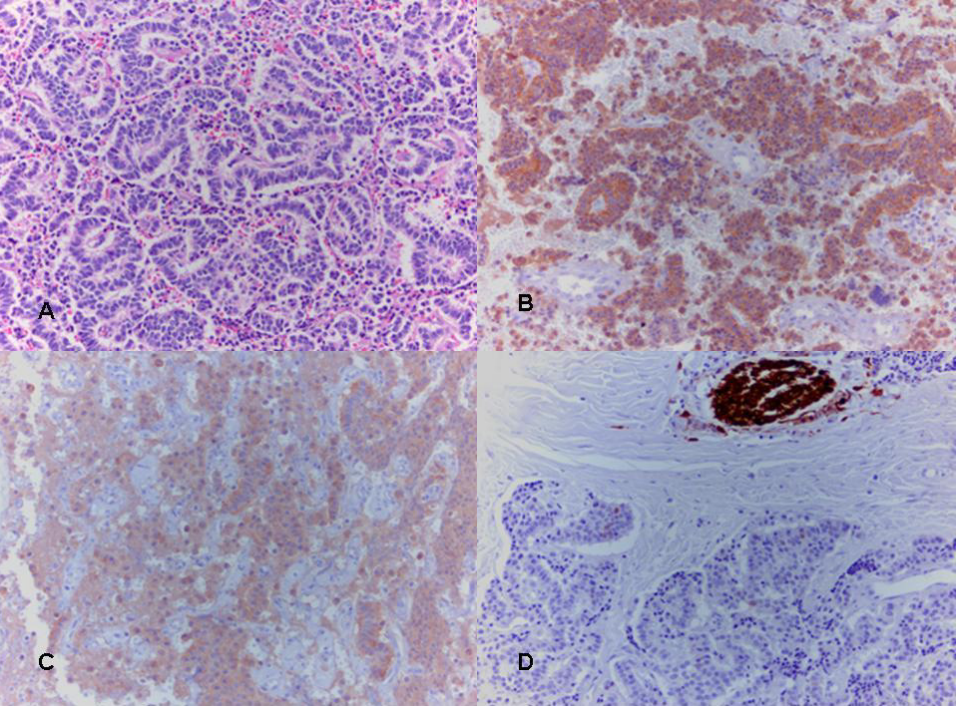
**H&E, synaptophysin, chromogranin and S-100 staining of the resected tumor**. A) H&E stain showing uniform cells arranged in chords. B) Synaptophysin and C) chromogranin staining were strongly positive. D) S-100 staining was negative (positive stain on the photograph represents a nerve which serves as an internal control).

The specimen consisted of a tumor mass with surrounding fibrous tissue. There was no evidence of lymphatic tissue in the specimen. Permanent sections revealed cytologically bland and monotonous cells arranged in cords or ribbons. Nuclei were round to oval, small, and occasionally featuring nucleoli. Mitotic rate was low, no necrosis or angioinvasion were noted. Characteristic "salt-and-pepper" neurosecretory granules were seen. Immunohistochemistry showed positivity for synaptophysin, neuron specific enolase, chromogranin and cytokeratin. The tumor was negative for S-100 (Figure 6). A paraganglioma was ruled out since the cells did not exhibit a nesting cell growth pattern. The cells were not large and did not have abundant cytoplasm. Intracytoplasmic globules were not present. The cells were positive for cytokeratin, while paragangliomas are usually negative. Most characteristically, S-100 staining is positive and highlights the sustentacular cells between the solid-sheets of a paraganglioma. The resected tumor was negative for S-100.

All of these features confirmed this tumor to be a well differentiated neuroendocrine tumor according to the WHO classification.

## Discussion

The presacral space is a region bounded by the rectum anteriorly, sacrum posteriorly, peritoneal reflection superiorly and perineal muscles inferiorly. The differential diagnosis of lesions arising in this area include solid tumors such as teratomas, chordomas, paragangliomas and ependymomas, or cystic lesions, such as tailgut cysts, duplication cysts, and dermoid cysts, thought to arise from the embryological tailgut or neurenteric canal. An association between tailgut cysts and presacral NETs is described in the literature. Furthermore, NETs of the presacral space are histopathologically similar to rectal NETs. The theory is that the presence of remnant hindgut cells may be the basis for development of neuroendocrine tumors in this region.

A review of the literature revealed 15 prior cases of primary presacral NET [2-14] (Table 1). There may be a female preponderance with these rare tumors as two-thirds of the cases have been seen in women. Seven previously reported cases were associated with tailgut cysts and two were associated with sacrococcygeal teratomas. Interestingly, we report the first case in the literature of this tumor arising in a patient who had a history of imperforate anus.

**Table 1:** Previous case reports describing the presence of a presacral carcinoid

**Case number**	**Author(reference)**	**Age/sex**	**Associated anomaly**
1	[3]	61/Male	Sacrococcygeal teratoma
2	[4]	35/Female	Presacral teratoma
3	[5]	18/Female	Tailgut cyst
4	[6]	61/Male	None
5	[7]	NA/Female	None
6	[2]	19/Female	Tailgut cyst
7	[2]	19/Female	None
8	[2]	21/Female	None
9	[8]	42/Female	None
10	[9]	69/Female	Tailgut cyst
11	[10]	52/Male	Tailgut cyst
12	[11]	68/Male	Tailgut cyst
13	[12]	41/Female	Tailgut cyst
14	[13]	NA/NA	Tailgut cyst
15	[14]	37/Male	Sacrococcygeal teratomas
16	This report	58/Female	Imperforate anus

Incomplete migration and/or fusion of the urorectal septum with the cloacal membrane lead to persistence of the cloaca and/or fistulae. Imperforate anus results from persistence of the dorsal cloacal membrane [15,16] and ventral persistence causes absent urethral and vaginal openings. There appears to be a 'malfunctioning of cells ingressing from an end-stage primitive streak' that leads to defective development of the dorsal cloaca [17]. Our postulate is that incomplete or impaired migration of hindgut cells led to both our patient's imperforate anus, and the predisposition to develop a primary neuroendocrine tumor from persistent hindgut rests in the presacral region. In fact, there was no evidence suggesting that the tumor emanated from the rectal wall. Colonoscopy failed to show a primary rectal tumor. EUS and CT images clearly demonstrate the tumor location outside the rectal wall. At surgery, the tumor was found to not involve the rectal wall. A somatostatin receptor scintography scan ruled out the chance that this tumor was metastatic from elsewhere as primary uptake was found in the presacral mass with no uptake in other sites. While carcinoid tumors can arise from other pelvic organs, besides the rectum, there was no evidence of primary tumors in the vagina, cervix or ovaries.

Our patient presented initially with abdominal pain and diarrhea, and was found to have an incidental presacral mass on CT scan. It was determined that her presenting symptoms were not related to the presence of this tumor but to a brief episode of nonspecific colitis. The neuroendocrine tumor was diagnosed after an appropriate diagnostic work-up following the most current guidelines [18,19]. Classically, gastroenteropancreatic neuroendocrine tumors are indolent and do not manifest symptoms unless they are either secretory tumors or are locally advanced to cause obstructive symptoms or have metastasized. The carcinoid syndrome, characterized by such symptoms as flushing, diarrhea, and wheezing, usually results from liver metastasis and subsequent release of vasoactive compounds such as serotonin [18]. The somatostatin receptor scintigraphy scan demonstrated no metastasis, nor did our patient exhibit a constellation of symptoms to suggest an actual carcinoid syndrome. Furthermore, she did not present with obstructive-type symptoms secondary to the mass. The histopathologic findings were of a well-delineated mass composed of highly differentiated neuroendocrine cells, displaying no angioinvasion and with a low mitotic index. The tumor cells were positive for chromogranin, synaptophysin, cytokeratin and negative for S-100. A paraganglioma was ruled out based on these findings and the lack of the typical histologic appearance. Our patient's clinical picture and above histopathologic findings are all consistent with the standard World Health Organization (WHO) classification of a well-differentiated gastroenteropancreatic neuroendocrine tumor of probable benign behavior [18,20,21].

In conclusion, we report the first case of primary presacral neuroendocrine tumor, of likely gastroenteropancreatic and, more specifically, originating from abnormal migration of hindgut cells in a patient with imperforate anus. There was no tumor involvement of the rectal wall on preop EUS or CT imaging or during surgical resection. The presence of previous surgery in the pelvis to create a neoanus resulted in increased complexity when determining the treatment options for this patient. However, as demonstrated by this case for tumors below the S3 level, despite previous surgery in the low pelvis, a transsacral approach for resection should be feasible, with careful preservation of the rectal wall [8].

## Competing interests

The author(s) declare that they have no competing interests.

## Authors' contributions

TK- Participated in the design of the study and helped to draft the manuscript. SRG- Conceived of the study and participated in its design and coordination and help to draft the manuscript. SNH- conceived of the study, participated in its design and coordination and helped to draft the manuscript, performed the revisions. All authors read and approved the final version of the manuscript.
